# Cerebral white matter lesions – associations with Aβ isoforms and amyloid PET

**DOI:** 10.1038/srep20709

**Published:** 2016-02-09

**Authors:** Danielle van Westen, Daniel Lindqvist, Kaj Blennow, Lennart Minthon, Katarina Nägga, Erik Stomrud, Henrik Zetterberg, Oskar Hansson

**Affiliations:** 1 Diagnostic Radiology, Department of Clinical Sciences Lund, Lund University; 2 Imaging and Function, Skåne University Health Care, Lund Sweden; 3 Psychiatry, Department of Clinical Sciences Lund, Lund University, Lund, Sweden; 4 Division of Psychiatry Skåne, Lund, Sweden; 5 Clinical Neurochemistry Laboratory, Institute of Neuroscience and Physiology, The Sahlgrenska Academy at University of Gothenburg, Mölndal, Sweden; 6 Clinical Memory Research Unit, Department of Clinical Sciences Malmö, Lund University, Sweden; 7 Memory Clinic, Skåne University Health Care, Malmö, Sweden; 8 Department of Molecular Neuroscience, UCL Institute of Neurology, Queen Square, London WC1N 3BG, United Kingdom

## Abstract

Small vessel disease (SVD) and amyloid deposition may promote each other, with a potential association between SVD and altered production or clearance of β-amyloid (Aβ) affecting its cleavage products. We investigated the relationship between SVD, multiple isoforms of Aβ in cerebrospinal fluid (CSF) and cortical Aβ in 831 subjects with cognitive performance ranging from normal to Alzheimer’s disease (AD) (the Swedish BioFINDER study). SVD was estimated as white matter lesions (WML) and lacunes. 18F-flutemetamol PET was performed in 321 subjects. Lower CSF levels of Aβ38 and Aβ40 were consistently associated with increased WML in all subgroups, while lower levels of CSF Aβ42 were associated with WML mainly in AD. CSF Aβ38 and Aβ40 were associated with regional WML in all regions, while CSF Aβ42 was associated with temporal WML only. A composite measure of 18F-flutemetamol uptake was not associated with WML, and regional 18F-flutemetamol uptake only with temporal WML. Lacunes were not associated with Aβ isoforms nor 18F-flutemetamol uptake. Our results suggest that WML may be associated with alterations in the production or clearance of Aβ species, particularly of Aβ38 and Aβ40. However, in AD cases, Aβ42 pathology might be associated with WML, especially in the temporal lobe.

While cerebral small vessel disease (SVD) affects perforating cerebral arterioles, capillaries and venules, the term SVD is also used to describe the resulting brain damage, comprising mainly subcortical lesions such as small infarcts, lacunes, white matter lesions (WML), enlarged perivascular spaces and microbleeds^1^. WML are the most common manifestation of SVD and especially abundant in elderly, but the suggested prevalence has varied substantially between different studies^2^,^3^,^4^. Cerebral microbleeds (MB) are markers of vascular pathology including cerebral amyloid angiopathy (CAA) and potentially have direct effects on brain function^5^. SVD will lead to a number of downstream effects including fibrosis impairing drainage of fluid and proteins from the extravascular space along the blood vessels^6^, with accumulation of proteins, including amyloid beta (Aβ)^7^,^8^.

Cleavage of the amyloid precursor protein (APP) by β- and γ-secretases yields Aβ peptides with Aβ38, Aβ40 and Aβ42 as the most common variants^9^. In most cells, Aβ38 and Aβ42 are produced at relatively low levels, typically 5–20% of the total Aβ detected, and the major species generated is Aβ40 that typically constitutes over 50% of total Aβ^10^,^11^. Aβ38, Aβ40 and Aβ42 differ in aggregation propensity: Aβ38 is soluble, present in the vasculature of in sporadic and familial AD patients and never found in senile plaques, Aβ40 is somewhat aggregation-prone, it is the main component for vascular amyloid and is often found in CAA, whereas Aβ42 is the most aggregation-prone, forming cortical fibrillar Aβ that will be deposited as amyloid plaques associated with Alzheimer’s disease^12^,^13^.

SVD and AD share common risk factors, such as hypertension during midlife, diabetes mellitus, smoking, the apolipoprotein E (*APOE*) ε4 allele and particularly age^14^,^15^. A two hit, potentially self-reinforcing mechanism has been proposed, suggesting that SVD may initiate amyloid deposition on the one hand, and amyloid deposition may increase the susceptibility to develop SVD on the other hand^16^,^17^. Increased knowledge about the role of Aβ species in SVD may improve treatment and prevention strategies for individuals with SVD, since accumulation of multiple vascular risk factors, especially in midlife, can substantially increase the risk for dementia^18^. This has been confirmed by the suggested declining trend in dementia risk, occurring in parallel with the decreasing incidence of cardiovascular events in high-income countries^19^,^20^. A recent study on the association between SVD and amyloid accumulation found higher WML to be associated with lower levels of CSF Aβ42 in vascular dementia and in subjective cognitive decline but not in AD, which was interpreted as a connection between SVD and AD pathology^21^. However, these findings may alternatively suggest that SVD is related to a general decrease in beta amyloid, reflecting a diminished secretion of amyloid precursor protein (APP), rather than the AD specific phenomenon of Aβ42 aggregation. If this were the case, WML would be associated with lower CSF levels of all amyloid species, including Aβ38 and Aβ40 in addition to Aβ42. Therefore, we readdressed the association between SVD and amyloid deposition including multiple markers of amyloidogenic APP-processing and Aβ deposition. Specifically, we studied the relationship between WML and lacunes assessed with neuroimaging and CSF levels of Aβ38, Aβ40 and Aβ42. In addition we measured amyloid uptake using 18F-flutemetamol PET, that reflects cortical levels of fibrillar Aβ associated with amyloid plaques. The study population consisted of 831 individuals from four diagnostic groups that differed in their cognitive performance, cognitively healthy elderly (CHE), and subjects with subjective cognitive decline (SCD), mild cognitive impairment (MCI) and AD; in order to confirm results in a separate cohort of subjects with a neurodegenerative disease, other than AD albeit associated with amyloid accumulation, a fifth diagnostic group, consisting of subjects with Parkinson’s disease (PD) without dementia, was also included.

## Results

### Demographics

Demographic data are given in Table 1. WML volume, total Fazekas score and total Age Related White Matter Changes (ARWMC) score were highly correlated, average partial correlation coefficient 0.806, *p* < 0.001, pooled data from the CHE, SCD, MCI and PD cohorts. CSF levels of Aβ38 and Aβ40 did not correlate with the composite SUVR for amyloid PET, partial correlation coefficient 0.027 and 0.088, respectively, *p* > 0.05. In contrast, the CSF level of Aβ42 showed a high inverse correlation with the composite SUVR for amyloid PET, partial correlation coefficient −0.668, *p* < 0.001, pooled data from the CHE, SCD and MCI cohorts. All correlation analyses were corrected for age, sex and hippocampal volume.

### Associations between total WML and CSF Aβ38, Aβ40 and Aβ42

In the CHE, SCD, MCI, and PD groups, lower CSF levels of Aβ38 and Aβ40 were significantly associated with increased WML volume (Table 2). However, there were no significant associations between CSF levels of Aβ42 and WML volume in any of the groups (Table 2). To confirm these results, visual assessment of WML using the Fazekas scale was performed, now including the AD group. Again, higher Fazekas scores (indicating more severe WML) was associated with lower CSF levels of Aβ38 and Aβ40 in the SCD and the MCI groups, and lower levels of Aβ38 in the PD group (Table 2). CSF Aβ42 was associated with WML only in the AD group, in addition to CSF Aβ38 and Aβ40 (Table 2).

Next, the subjects where ^18^F-flutemetamol PET was performed, namely the CHE, SCD and MCI cases, were pooled to perform subgroup analyses. Lower CSF levels of Aβ38 and Aβ40 were significantly and consistently associated with increased WML volume in all participants (Table 3). The relationship between WML volume and CSF levels of Aβ42 was stronger in *APOE* ε4 negative than *APOE* ε4 positive participants (Table 3). Lower levels of CSF Aβ42 were also associated with increased WML volume, but at a lower magnitude than Aβ38 and Aβ40 (Table 3).

### Associations between total WML and amyloid PET

We also used amyloid PET imaging with the ^18^F-flutemetamol ligand to study the cortical levels of Aβ fibrils in CHE, SCD and MCI. The ^18^F-flutemetamol composite score was not significantly associated with WML volume or Fazekas score in any of the studied groups (Table 3).

### Associations between regional distribution of WML and CSF Aβ species or amyloid PET

The potential regional impact of SVD was assessed from WML in various brain regions using the ARWMC scale. Lower levels of CSF Aβ38 and Aβ40 were significantly associated with increased WML in the frontal, parieto-occipital and temporal lobes, but not in infratentorial regions (Table 4). Lower levels of CSF Aβ42 and higher levels of the composite ^18^F-flutemetamol uptake were significantly associated with WML in the temporal lobe only (Table 4). Since regional ARWMC scores correlated significantly with the total ARWMC, analysis was redone with the total ARWMC included as a predictor. Again, only the association between CSF Aβ42 and temporal ARWMC was significant.

When examining the relationship between regional WML and regional ^18^F-flutemetamol uptake, increased ^18^F-flutemetamol uptake in frontal, parietal, temporal, occipital regions were all associated with a higher temporal ARWMC score (Table 5).

### Lack of association between lacunes and CSF Aβ species or amyloid PET

Neither CSF Aβ42 nor the composite ^18^F-flutemetamol uptake were associated with the presence of lacunes in any group (Table 3, results from CHE, SCD, MCI groups).

## Discussion

Our main findings are firstly that more pronounced WML is strongly and consistently associated with lower levels of CSF Aβ38 and Aβ40, while the association with CSF Aβ42 levels is weak or absent. This pattern was found in all diagnostic groups, except for the AD group where WML were associated with all three Aβ isoforms. Secondly, the composite ^18^F-flutemetamol SUVR, that reflects cortical fibrillar Aβ deposition and strongly correlated with CSF Aβ42, was not associated with WML in any of the CHE, SCD and MCI groups (PET data unavailable for the PD and AD groups).

Two hypotheses might explain this consistent, negative relationship between WML and Aβ38 and Aβ40. Firstly, reduced clearance of Aβ38 and Aβ40 from the brain parenchyma may enhance deposition of these Aβ species in the cerebral vessel walls, as has been reported for Aβ40^11^,^22^. Evidence favors the hypothesis that amyloid fibrils deposited in the vessel wall and senile plaques differ from each other, as the major species in CAA is Aβ39 or Aβ40, and in senile plaque Aβ42, although Aβ40 may also be present^23^,^24^. Similarly, Aβ38 seems to be predominantly located within the vasculature in postmortem brains of sporadic and familial AD patients^11^,^25^. Impaired vascular clearance of Aβ across the BBB and increased Aβ brain capillary deposition has been reported in transgenic mice producing low levels of the poorly cleared Dutch/Iowa mutant forms of Aβ, which are vasculotropic and rich in β-sheets^26^. β-amyloid deposition, that is directly toxic to smooth muscle cells, leads to constriction of cerebral blood vessels, and thus to perturbation of cerebral perfusion, loss of homeostasis of the neuronal environment, ultimately leading to ischemia and consequently WML^27^,^28^. Also the previously reported positive association between the Aβ40 level in plasma and WMH in subjects with MCI, AD and CAA, suggests that circulating Aβ40 is a potential contributor to microvascular dysfunction^29^. Reduced clearance may play also a role in CAA that is associated with vascular deposition of Aβ40 and Aβ42 with Aβ40 as the major isoform. Decreased levels of CSF Aβ40 as well as Aβ42 have been reported in CAA patients, suggesting that Aβ40 and Aβ42 may be trapped in the cerebral vasculature and escape the drainage pathways that otherwise transport amyloid β proteins toward the cerebrospinal fluid^30^. It has been hypothesized that SVD may exacerbate CAA by promoting cerebral edema that needs to compete with Aβ for clearance via perivascular drainage pathways^31^. A larger prevalence of WML in CAA compared to AD and MCI has been reported with WMH correlating with plasma levels of Aβ40^29^.

Secondly, SVD, and especially WML, may be associated with reduced production of Aβ in the brain. An inverse relationship between the volume of WML and CSF APP metabolites (including Aβ38, Aβ40 and Aβ42) in both stroke patients and SCD/MCI patients has been reported^32^. In that study, lower levels of CSF APP metabolites in the stroke group compared to the SCD/MCI-group suggested that ischemia influences APP metabolism, probably through inhibition of fast axonal transport of APP. This is consistent with evidence from a combined animal and neuropathological study that acute ischemic lesions can trigger accelerated amyloid deposition, most likely through interference with amyloid clearance pathways^33^. White matter pathology underlying WML has been suggested to affect neuronal activity by a body of clinical work reporting a direct relationship between white matter integrity and cognitive performance^34^. In addition, a negative correlation has been reported between WML volume and connectivity strength in regions with decreased connectivity in MCI patients as compared to controls^35^. Notably, the production of Aβ and its secretion into the extracellular space are tightly regulated by neuronal activity *in vitro* and *in vivo*; increased neuronal activity enhances Aβ production, and blocking neuronal activity has the opposite effect^36^.

An inverse association between WML and AD specific CSF Aβ42 has recently been reported in subjects with MCI^21^. Even though we reproduced this association in the pooled CHE, SCD and MCI data (Table 3), our results showed that the association with WML was much stronger for Aβ38 and Aβ40, that were not investigated in the study by Kester *et al.*^21^ We confirm this by the lack of association between WML and ^18^F-flutemetamol PET imaging, that reflects fibrillar Aβ deposition. We found that only in cases with AD dementia, Aβ42 pathology might be associated with WML, especially in the temporal lobes. Previous smaller scale studies in subjects with cognitive status ranging from normal to mild dementia have reported a similar lack of association between WML and amyloid PET^37^,^38^,^39^,^40^. Thus, non-AD-specific subcortical changes may affect global levels of Aβ isoforms in the central nervous system^32^. It should be noted that amyloid ligands such as ^18^F-flutemetamol were developed to bind to aggregated Aβ. For example, the Pittsburgh compound B (PiB) ligand may bind to vascular amyloid^41^,^42^ Higher PIB uptake has been consistently reported in non-demented CAA patients, at a level intermediate between healthy controls and AD^43^,^44^.

Parenchymal deposition of Aβ protein and lower CSF levels of Aβ42 are associated with increased risk for AD development. In addition, vascular deposition of Aβ may also be a primary driver of AD and affect the production and clearance of APP. Thus increased WML burden among patients with AD might reflect accumulation of vascular Aβ to some degree. Interestingly, we found a significant association between WML and CSF Aβ42 in the AD dementia group, indicating that Aβ42-related plaque pathology might be associated with SVD in individuals with dementia due to AD.

Amyloid PET-uptake and WML volume have been shown to independently predict AD diagnosis and thus WML may be involved in the clinical manifestation of AD^38^. WML are more prevalent and severe in AD patients compared with nondemented adults matched for demographic characteristics and brain regions where WML are most severe in AD, reportedly occur in the same location as AD pathology and areas showing the greatest metabolic dysfunction in AD^45^. It cannot be ruled out that some of the subjects with clinically diagnosed AD may actually suffer from mixed dementia, with both AD and vascular (i.e. WML) contributions^46^. While this may affect the associations with amyloid species presently found in this group, the total Fazekas and ARWMC scores in the AD group were comparable to those in the CHE, SCD and PD groups and lower than in the MCI group.

Our main finding that the much stronger association between WML and CSF Aβ38 and Aβ40, than CSF Aβ42 was confirmed in the PD group. This strengthens previous evidence suggesting that also PD should be targeted by treatment for vascular risk factors since comorbid WML are common in PD and affect motor function and cognition^47^,^48^ We did not assess the impact of the interplay between amyloid and SVD on cognition. According to a recent study in non-demented elderly amyloid and vascular pathologies seemed to be at least partly independent processes that both affect longitudinal cognitive trajectories adversely and are major drivers of cognitive decline in the elderly^49^. This further stresses the need for treatment of risk factors for SVD in neurodegenerative disease.

We acknowledge limitations in the present study. Firstly, some data are lacking, as for example ^18^F-flutemetamol PET for the PD and AD cases and quantitative WML analysis for AD cases; in the CHE, SCD and MCI groups, ^18^F-flutemetamol PET was available for 321 subjects (Table 1). Also, there is some heterogeneity in the imaging data since mainly CT images were available for the AD group. Data on cerebral microbleeds as marker of cerebral amyloid angiopathy are not included, since susceptibility weighted images (SWI) to assess their presence were available for the PD group. Secondly, the use of linear regression for ordinal scales as the Fazekas and ARWMC scales, may be questioned since the number of categories is limited, and the underlying model assumptions (linear change with each unit change in category) may not be valid. Data from these scales were included since the WML volume could not be determined in the AD group; in addition, these scales have been used in other studies, for example the study by Kester *et al.*^14^ Instead, the WML volume provides a continuous variable and the lack of significant association between the Fazekas score and CSF Aβ species in the CHE and PD groups, where WML were less abundant, should be explained by the inherent differences in these two variables. Thirdly, lacunes, also markers of SVD, showed a significant association with CSF Aβ38 and CSF Aβ40 only in the MCI group. Lacunes and WML differ regarding their pathogenesis, with lacunes being due to sudden, total occlusion of an end artery, whereas white matter lesions are associated with longstanding, less severe general hypoperfusion of brain tissue. The consequences of lacunes and WML with regard to amyloid production and clearance are also likely to differ, with the former resulting in severe, but focal damage to the brain parenchyma in a smaller area, and the latter in less severe, but more widespread damage. In addition, the low prevalence of lacunes in the CHE and SCD groups may have reduced statistical power.

In conclusion, our findings suggest that WML are associated with lower CSF Aβ38 and Aβ40 levels, probably reflecting lower overall Aβ production due to either decreased expression or secretase processing of APP. This is confirmed by the lack of association between WML and ^18^F-flutemetamol PET, that reflects fibrillar Aβ deposition. However, in cases with AD dementia Aβ42 pathology might be associated with WML, especially in the temporal lobes.

## Methods

### Ethical approval and patient consent

The study protocol was designed in accordance with guidelines outlined in the Declaration of Helsinki and approved by the Research Ethics Committee at Lund University, Lund, Sweden. Written informed consent was obtained from all participants.

### Study population

The first sample, including cognitively healthy elderly (CHE) subjects and subjects with mild cognitive symptoms (MCS), i.e. patients with either subjective cognitive decline (SCD) or objective impairment (MCI), was part of the prospective and longitudinal Swedish BioFINDER (Biomarkers For Identifying Neurodegenerative Disorders Early and Reliably) study (http://www.biofinder.se). The 267 CHE participants, recruited from the population-based Malmö Diet Cancer study^50^, were eligible for inclusion in the cognitively healthy elderly cohort of the Swedish BioFinder study if they 1) were aged ≥60 years old, 2) scored 28–30 points on Mini-Mental State Examination (MMSE), 3) did not suffer from any subjective cognitive impairment and 4) were fluent in Swedish^51^. Exclusion criteria included presence of significant neurologic disease, severe psychiatric disease, dementia or MCI. The 360 MCS cases complaints consisted of patients enrolled consecutively at three memory outpatient clinics in Sweden. They were thoroughly assessed by physicians with special interest in dementia disorders. The inclusion criteria were: 1) referred to the memory clinics because of cognitive impairment; 2) not fulfilling the criteria for dementia; 3) a MMSE score of 24–30 points; 4) age 60–80 years and; 5) fluent in Swedish. Exclusion criteria were: 1) cognitive impairment that without doubt could be explained by a condition other than prodromal dementia; 2) severe somatic disease, and; 3) refusing lumbar puncture or neuropsychological investigation. MCS patients were assessed based on a neuropsychological battery assessing four broad cognitive domains including verbal ability, visuospatial construction, episodic memory, and executive functions. A senior neuropsychologist then stratified all patients into those with SCD (no measurable cognitive deficits) or MCI according to the consensus criteria for MCI suggested by Petersen^52^. In total 165 patients (46%) were classified as SCD and 195 (54%) patients as MCI.

The second sample here referred to as the ‘AD cohort’, comprised 110 subjects with AD. Subjects were recruited from a retrospective study performed between 2008 and 2014. All patients met the dementia criteria and were diagnosed as probable AD according to NINCDS-ADRDA^53^ and to have CSF Aβ42 below 550 ng/L to confirm the presence of amyloid pathology.

The third sample, comprising 89 PD cases without dementia, here referred to as the “PD cohort”, is also part of the prospective Swedish BioFINDER study. PD diagnosis was set according to the NINDS Diagnostic Criteria^54^.

Demographic characteristics of the participants are presented in Table 1. More information is given in the Supplementary information online and at www.biofinder.se.

### Image acquisition and assessment of cerebrovascular disease

In CHE, SCD, MCI and PD subjects, MR imaging was performed at 3 T systems and included trasnversal T2 FLAIR and high resolution isotropic MPRAGE. In AD subjects, imaging data included axial CT images in 96 cases and axial FLAIR images acquired at 1.5 T MR systems in 14 cases.

WML and lacunes assessed from MRI, and WML from CT were used as markers of SVD. For MR data in the CHE, SCD, MCI and PD subjects, automated segmentation of WML using the LST toolbox implemented in SPM8, generated a total lesion volume [mL], here named ‘WML volume’, for each individual^55^. Visual rating of WML on FLAIR images according to the Fazekas scale^56^, resulted in a total Fazekas score, and according to the ARWMC scale^57^, resulting in regional as well as total scores. CT images in the AD cohort were assessed for WML according to the Fazekas scale. Scores from the left and right hemispheres were summarized for statistical analysis. The presence of lacunes was assessed on FLAIR and MPRAGE images according to Wardlaw^58^. This variable was dichotomized as lacunes, present or absent. More information is given in the Supplementary Methods online.

### CSF collection and analysis

The collection procedure and analysis of CSF followed the Alzheimer’s Association Flow Chart for CSF biomarkers^59^. Lumbar CSF samples were collected and stored in polypropylene tubes at −80 °C and analyzed in one batch. CSF levels of Aβ38 and Aβ40 were measured using EUROIMMUN ELISAs (EUROIMMUN AG, Lübeck, Germany), and CSF Aβ42 and CSF P-tau were measured using INNOTEST ELISAs (Fujirebio Europe, Gent, Belgium).

### ^18^F-Flutemetamol PET imaging and analysis

In 122 CHE, 101 SCD and 98 MCI subjects, the cerebral Aβ burden was measured using [^18^F]-flutemetamol PET. Subjects received a single dose of [^18^F]flutemetamol and its average uptake was estimated from sum images acquired 90–110 min post injection^60^. Image processing was performed as previously described^61^. The standardized uptake value ratio (SUVR) was determined as the regional and composite tracer uptake normalized for the mean uptake in the cerebellar cortex, which is free of fibrillar plaques. More information is given in the Supplementary Methods online.

### Statistics

Statistics were computed using SPSS version 22 (IBM). Non-normally distributed variables were transformed into normality using log transformation. Group-wise comparisons of baseline characteristics were performed using the Pearson Chi-square test for categorical variables and ANOVA for continuous variables. Since WML rating in the AD group mainly was performed on CT images, WML scores were not compared between the AD group and the other groups.

Regression analyses were performed to test the associations between estimates of SVD and amyloid deposition, with WML volume, Fazekas and ARWMC scores and lacunes as dependent variables and CSF-biomarkers and PET composite scores as independent variables. Regressions were conducted separately for each independent variable. Linear regression was used for continuous (WML volume, Fazekas and ARWMC scores) and logistic regression for binary variables (lacunes). All regression analyses were adjusted for age, sex, and hippocampal volume. Hippocampal volume was included as a co-variate in the regression analyses to adjust for AD disease stage as suggested by Kester *et al.*^21^ The values in Table 2, 3, 4 and 5 represent standardized betas from linear regression in each group as specified in the first column. Since four markers of amyloid deposition were studied, only p-values < 0.0125 should be considered significant according to Bonferroni-adjustment. Comparison of the demographic data in Table 1 involved comparison of each diagnostic group to each of the other groups; application of Bonferroni correction would result in a significance level of p-values < 0.005 as indicated.

## Additional Information

**How to cite this article**: van Westen, D. *et al.* Cerebral white matter lesions – associations with Aβ isoforms and amyloid PET. *Sci. Rep.* **6**, 20709; doi: 10.1038/srep20709 (2016).

## Supplementary Material

Supplementary Information

## Figures and Tables

**Table 1:** Baseline characteristics by clinical diagnosis.

	Cognitively healthy elderly (n = 267)	Subjective cognitive deficit (n = 165)	Mild cognitive impairment (n = 195)	PD patients (n = 89)	AD patients (n = 110)
Age (mean + SD)	72.9 + 5.0	70.0 + 5.8a***	71.2 + 5.6a***	65.4 + 11.2a,b, c***	74.5 + 7.3a*,b, c, d***
Proportion female (%)	61.4	57.6	44.6a***, b*	38.2a,b***	62.7c, d***
*APOE* ε4 carriers (%)	30.7	38.5	47.9a***	N/A	67.3a,b*** c***
WML volume					
(median, IQR)	5.3, 2.0–14.2	6.5, 1.7–18.9	12.5, 4.5–34.0a,b***	5.0, 1.5–16.4c***	N/A
Fazekas total score (median, IQR)	4.0, 2.0–6.0	5.0, 3.0–7.0	6.0, 3.0–10.0a,b***	4.0, 1.0–7.5b*, c***	4.0, 2.0–7.0c***
AWMRC total (median, IQR)	4.0, 1.0–8.0	5.0, 2.0–8.0	6.0, 2.0–10.0a***, b*	4.0, 0.0–7.50c*	3.5, 0.8–8.0c***
Lacunes (%)	5.7	8.1	11.3a*	2.2c*	3.6c*
CSF Aβ38 (median, IQR, pg/mL)	1727.5, 1492.3–2011.0	1797.0, 1432.3–2065.3	1646.5, 1357.0–1889.3a***, b*	1626.0, 1310.0–1841.0a,b***	1649.0, 1360.8–1935.8a, b***
CSF Aβ40 (median, IQR, pg/mL)	4549.0, 3515.5–5483.5	4837.5, 3514.3–6298.8	4339.0, 3427.8–5546.0b*	3757.0, 2996.5–4784.0a, b, c***	3826.5, 3120.5–5159.8a, b***, c**
CSF Aβ42 (median, IQR, pg/mL)	665.0, 509.8–778.0	657.0, 440.9–802.8	468.0, 351.0–711.0a,b***	644.0, 481.5–766.5c***	365.0, 297.8–428.8a, b, c, d***
Composite PET SUVR (median, IQR)	1.24, 1.12–1.34 (n = 122)	1.23,1.13–1.68 (n = 101)a*	1.55, 1.20–2.09 (n = 98)a, b***	N/A	N/A

^a^vs cognitively healthy elderly.

^b^vs subjective cognitive deficit.

^c^vs mild cognitive impairment.

^d^vs PD. ^•^p < 0.05, **p < 0.01, ***p < 0.005

Only p-values < 0.005. should be considered significant according to Bonferroni-adjustment.

**Table 2:** Associations between cerebrovascular disease, Aβ isoforms and amyloid PET per diagnostic group.

Group	Parameter	WML volume ± 95% CI			Fazekas score ± 95% CI			Lacunes ± 95% CI		
Cognitively	CSF Aβ38	−0.151**	−0.260	−0.042	−0.071	−0.191	0.048	−0.254	−1.369	0.861
healthy	CSF Aβ40	−0.118*	−0.228	−0.008	−0.058	−0.178	0.061	−0.363	−1.940	1.213
elderly	CSF Aβ42	−0.075	−0.185	0.036	0.017	−0.103	0.137	−0.795	−2.406	0.815
	Composite SUVR	0.037	−0.137	0.210	−0.057	−0.241	0.127	0.570	−2.911	4.052
										
Subjective	CSF Aβ38	−0.211**	−0.341	−0.081	−0.170*	−0.312	−0.028	2.306	−0.317	−0.030
cognitive	CSF Aβ40	−0.243***	−0.371	−0.115	−0.197**	−0.337	−0.057	1.144	−0.309	−0.020
deficit	CSF Aβ42	−0.079	−0.213	0.055	−0.066	−0.211	0.079	0.976	−0.154	0.142
	Composite SUVR	0.099	−0.075	0.273	0.070	−0.118	0.257	−0.686	−0.187	0.231
										
Mild	CSF Aβ38	−0.264***	−0.396	−0.133	−0.174*	−0.317	−0.030	−1.947*	−3.655	−0.239
cognitive	CSF Aβ40	−0.286***	−0.417	−0.155	−0.164*	−0.309	−0.020	−1.018	−2.312	0.276
impairment	CSF Aβ42	0.000	−0.138	0.138	−0.006	−0.154	0.142	0.715	−0.501	1.931
	Composite SUVR	−0.028	−0.226	0.170	0.022	−0.187	0.231	0.149	−2.755	3.052
										
PD−patients	CSF Aβ38	−0.181**	−0.310	−0.053	−0.188*	−0.365	−0.011	NA		
	CSF Aβ40	−0.185**	−0.314	−0.056	−0.175	−0.225	−0.125	NA		
	CSF Aβ42	−0.066	−0.204	0.072	0.010	0.386	−0.366	NA		
										
AD−patients	CSF Aβ38	NA			−0.258*	−0.447	−0.070	NA		
	CSF Aβ40	NA			−0.299**	−0.485	−0.113	NA		
	CSF Aβ42	NA			−0.225*	−0.413	−0.038	NA		

Values represent standardized beta (linear regression, WML volume and total Fazekas score) and Beta (logistic regression, lacunes) with 95% confidence intervals; prior to analysis, the natural logarithm of parameters was calculated where appropriate in order to ensure normal distribution. Values are corrected for age, gender and hippocampal volume; the latter was determined using Adaboost, the mean value of the left and right hippocampus was normalized using the scaling factor provided by this software to account for head size differences.

In the PD and AD groups, 2.3% and 3.6% of the subjects had lacunes and the association between CSF Aβ species and lacunes was not analyzed. Data on WML volume were not available for the AD group, where imaging mainly was performed using CT.

*p < 0.05, **p < 0.0125, ***p > 0.001.

Only p−values < 0.0125 should be considered significant according to Bonferroni−adjustment.

**Table 3:** Associations between SVD, estimated as the volume of white matter lesions (WML), the total score of WML according to Fazekas, and lacunes, and amyloid isoforms in CSF and amyloid PET, in the pooled CHE, SCD and MCI subgroups.

Group	Parameter	WML volume ± 95% CI			Fazekas ± 95% CI			Lacunes ± 95% CI		
All CHE, SCD	CSF Aβ38	−0.219***	−0.288	−0.15	−0.150***	−0.227	−0.073	−0.841	−1.966	0.284
and MCI	CSF Aβ40	−0.205***	−0.274	−0.136	−0.128***	−0.205	−0.051	−0.418	−1.253	0.417
	CSF Aβ42	−0.086*	−0.158	−0.014	−0.055	−0.135	0.025	0.298	−0.513	1.109
	Composite SUVR	0.074	−0.031	0.179	0.071	−0.044	0.186	−0.12	−1.723	1.483
										
APOE ε4 −	CSF Aβ38	−0.282***	−0.394	−0.17	−0.203**	−0.328	−0.078	−2.354**	−4.104	−0.604
positive	CSF Aβ40	−0.266***	−0.381	−0.071	−0.154*	−0.277	−0.031	−1.544*	−2.879	−0.209
CHE, SCD	CSF Aβ42	−0.127*	−0.247	−0.007	−0.09	−0.221	0.041	0.971	−0.278	2.22
and MCI	Composite SUVR	0.153	−0.031	0.337	0.103	−0.105	0.311	−1.128	−3.842	1.598
										
APOE ε4 −	CSF Aβ38	−0.187***	−0.276	−0.098	−0.125*	−0.224	−0.026	0.229	−1.292	1.75
negative	CSF Aβ40	−0.174***	−0.263	−0.085	−0.117*	−0.216	−0.018	0.314	−0.776	1.404
CHE, SCD	CSF Aβ42	−0.128**	−0.22	−0.036	−0.086	−0.188	0.016	0.054	−1.275	1.383
and MCI	Composite SUVR	0.08	−0.052	0.213	0.06	−0.084	0.204	0.432	−1.969	2.833

Values represent standardized beta (linear regression, WML volume and total Fazekas score) and Beta (logistic regression, lacunes) with 95% confidence intervals; prior to analysis, the natural logarithm of parameters was calculated where appropriate in order to ensure normal distribution. Values are corrected for age, gender and hippocampal volume; the latter was determined using Adaboost, the mean value of the left and right hippocampus was normalized using the scaling factor provided by this software to account for head size differences.

*p < 0.05, **p < 0.0125, ***p > 0.001.

Only p-values < 0.0125 should be considered significant according to Bonferroni-adjustment.

**Table 4:** Associations between regional cerebrovascular disease, Aβ isoforms and amyloid PET, pooled data from the CHE, SCD and MCI groups.

Group	Parameter	ARWMC total	ARWMC frontal	ARWMC parieto- occipital	ARWMC temporal	ARWMC infratententorial
**CHE, SCD, MCI**	CSF Aβ38	−0.142***	−0.130***	−0.142***	−0.155***	−0.013
	CSF Aβ40	−0.121**	−0.111**	−0.122**	−0.137**	0.019
	CSF Aβ42	−0.040	−0.026	−0.073	−0.088*	0.031
	Composite SUVR	0.094	0.088	0.084	0.136*	0.015

SVD was estimated as the total and regional white matter lesion score according to the ARWMC scale. Values represent standardized beta (linear regression, ARWMC score) with 95% confidence intervals; prior to analysis, the natural logarithm of the CSF and PET parameters was calculated to ensure normal distribution. Values are corrected for age, sex and hippocampal volume; the latter was determined using Adaboost, the mean value of the left and right hippocampus was normalized using the scaling factor provided by this software to account for head size differences. *p < 0.05, **p < 0.0125, ***p > 0.001.

Only p-values < 0.0125 should be considered significant according to Bonferroni-adjustment.

**Table 5:** Associations between regional cerebrovascular disease and regional amyloid uptake measured as PET SUVR for CHE, SCD and MCI subjects.

Group	Parameter	ARWMC frontal			ARWMC parito-occipital			ARWMC temporal		
**CHE, SCD, MCI**	Frontal SUVR	0.088	−0.027	0.203	0.079	−0.036	0.194	0.140*	0.022	0.259
	Parietal SUVR	0.119	0.004	0.233	0.117	0.002	0.231	0.168**	0.050	0.285
	Occipital SUVR	0.097	−0.018	0.213	0.092	−0.023	0.207	0.148*	0.029	0.266
	Temporal SUVR	0.061	−0.053	0.175	0.055	−0.059	0.168	0.104	−0.014	0.221

Values represent standardized beta from linear regression with regional ARWMC as dependent and regional amyloid uptake as independent variable as well as the confidence intervals, and are corrected for age, sex and hippocampal volume. Cerebrovascular disease was estimated as the total score of white matter lesion rating according to the ARWMC scale, total sum, and regional, mean of left and right. PET SUVR was estimated the regional uptake, mean of left and right; the natural logarithm of PET estimates was used.

*p < 0.05, **p < 0.0125, ***p > 0.001.

Only p-values < 0.0125 should be considered significant according to Bonferroni-adjustment.

## References

[b1] Pantoni L. Cerebral small vessel disease: from pathogenesis and clinical characteristics to therapeutic challenges. Lancet Neurol. 9, 689–701 (2010). 20610345 10.1016/S1474-4422(10)70104-6

[b2] Ylikoski A. *et al.* White matter hyperintensities on MRI in the neurologically nondiseased elderly. Analysis of cohorts of consecutive subjects aged 55 to 85 years living at home. Stroke. 26, 1171–1177 (1995). 7604409 10.1161/01.str.26.7.1171

[b3] Breteler M. M. *et al.* Cerebral white matter lesions, vascular risk factors, and cognitive function in a population-based study: the Rotterdam Study. Neurology 44, 1246–1252 (1994). 8035924 10.1212/wnl.44.7.1246

[b4] Longstreth W. T. *et al.* Clinical correlates of white matter findings on cranial magnetic resonance imaging of 3301 elderly people. The Cardiovascular Health Study. Stroke. 27, 1274–1282 (1996). 8711786 10.1161/01.str.27.8.1274

[b5] Greenberg S. M. *et al.* Microbleed Study Group. Cerebral microbleeds: a guide to detection and interpretation. Lancet Neurol. 8, 165–174 (2009). 19161908 10.1016/S1474-4422(09)70013-4 PMC3414436

[b6] Weller R. O., Hawkes C. A., Kalaria R. N., Werring D. J. & Carare R. O. White matter changes in dementia: role of impaired drainage of interstitial fluid. Brain Pathol. 25, 63–78 (2015). 25521178 10.1111/bpa.12218 PMC8028907

[b7] Grinberg L. T. & Thal D. R. Vascular pathology in the aged human brain. Acta Neuropathol. 119, 277–290 (2010). 20155424 10.1007/s00401-010-0652-7 PMC2831184

[b8] Utter S. *et al.* Cerebral small vessel disease-induced apolipoprotein E leakage is associated with Alzheimer disease and the accumulation of amyloid beta-protein in perivascular astrocytes. J Neuropathol Exp Neurol. 67, 842–856 (2008). 18716559 10.1097/NEN.0b013e3181836a71

[b9] Czirr E. *et al.* Independent generation of Abeta42 and Abeta38 peptide species by gamma-secretase. J Biol Chem. 283, 17049–17054 (2008). 18426795 10.1074/jbc.M802912200

[b10] Wang R., Sweeney D., Gandy S. E. & Sisodia S. S. The profile of soluble amyloid beta protein in cultured cell media. Detection and quantification of amyloid beta protein and variants by immunoprecipitation-mass spectrometry. J Biol Chem. 271, 31894–31902 (1996). 8943233 10.1074/jbc.271.50.31894

[b11] Suzuki N. *et al.* An increased percentage of long amyloid beta protein secreted by familial amyloid beta protein precursor (beta APP717) mutants. Science. 264, 1336–1340 (1994). 8191290 10.1126/science.8191290

[b12] Ozawa K. 1. *et al.* Enhanced Abeta40 deposition was associated with increased Abeta42-43 in cerebral vasculature with Dutch-type hereditary cerebral hemorrhage with amyloidosis (HCHWA-D). Ann N Y Acad Sci. 977, 149–154 (2002). 12480745 10.1111/j.1749-6632.2002.tb04810.x

[b13] Portelius E. *et al.* Mass spectrometric characterization of brain amyloid beta isoform signatures in familial and sporadic Alzheimer’s disease. Acta Neuropathol. 120, 185–193 (2010). 20419305 10.1007/s00401-010-0690-1 PMC3568930

[b14] Casserly I. & Topol E. Convergence of atherosclerosis and Alzheimer’s disease: inflammation, cholesterol, and misfolded proteins. Lancet. 363, 1139–1146 (2004). 15064035 10.1016/S0140-6736(04)15900-X

[b15] Meng X. F. *et al.* Midlife vascular risk factors and the risk of Alzheimer’s disease: a systematic review and meta-analysis. J Alzheimers Dis. 42, 1295–1310 (2014). 25024338 10.3233/JAD-140954

[b16] Smith E. E. & Greenberg S. M. Beta-amyloid, blood vessels, and brain function. Stroke. 40, 2601–2606 (2009). 19443808 10.1161/STROKEAHA.108.536839 PMC2704252

[b17] Zlokovic B. V. Neurovascular pathways to neurodegeneration in Alzheimer’s disease and other disorders. Nat Rev Neurosci. 12, 723–738 (2011). 22048062 10.1038/nrn3114 PMC4036520

[b18] Qiu C. & Fratiglioni L. A major role for cardiovascular burden in age-related cognitive decline. Nat Rev Cardiol. 12, 267–277 (2015). 25583619 10.1038/nrcardio.2014.223

[b19] Qiu C. *et al.* Twenty-year changes in dementia occurrence suggest decreasing incidence in central Stockholm, Sweden. Neurology. 80, 1888–94 (2013). 23596063 10.1212/WNL.0b013e318292a2f9

[b20] Schrijvers E. M. *et al.* Is dementia incidence declining? Trends in dementia incidence since 1990 in the Rotterdam Study. Neurology. 78, 1456–63 (2012). 22551732 10.1212/WNL.0b013e3182553be6

[b21] Kester M. I. *et al.* Associations between cerebral small-vessel disease and Alzheimer disease pathology as measured by cerebrospinal fluid biomarkers. JAMA Neurol. 71, 855–862 (2014). 24818585 10.1001/jamaneurol.2014.754

[b22] McGowan E. *et al.* Amyloid phenotype characterization of transgenic mice overexpressing both mutant amyloid precursor protein and mutant presenilin 1 transgenes. Neurobiol Dis. 6, 231–244 (1999). 10448051 10.1006/nbdi.1999.0243

[b23] De la Torre J. C. Alzheimer disease as a vascular disorder: nosological evidence. Stroke. 33, 1152–1162 (2002). 11935076 10.1161/01.str.0000014421.15948.67

[b24] Prelli F., Castano E., Glenner G. G. & Frangione B. Differences between vascular and plaque core amyloid in Alzheimer’s disease. J Neurochem. 51, 648–651 (1988). 3292706 10.1111/j.1471-4159.1988.tb01087.x

[b25] Reinert J. *et al.* Aβ38 in the brains of patients with sporadic and familial Alzheimer’s disease and transgenic mouse models. J Alzheimers Dis. 39, 871–881 (2014). 24305500 10.3233/JAD-131373

[b26] Davis J. 1. *et al.* Early-onset and robust cerebral microvascular accumulation of amyloid beta-protein in transgenic mice expressing low levels of a vasculotropic Dutch/Iowa mutant form of amyloid beta-protein precursor. J Biol Chem. 279, 20296–20306 (2004). 14985348 10.1074/jbc.M312946200

[b27] Hawkes C. A. *et al.* Disruption of arterial perivascular drainage of amyloid-beta from the brains of mice expressing the human APOE epsilon4 allele. PLoS One. 7, e41636 (2012). 22848551 10.1371/journal.pone.0041636 PMC3404985

[b28] Hawkes C. A., Carare R. O. & Weller R. O. Amyloid and tau in the brain in sporadic Alzheimer’s disease: defining the chicken and the egg. Acta Neuropathol. 127, 617–618 (2014). 24452628 10.1007/s00401-014-1243-9 PMC3950602

[b29] Gurol, M. E1. *et al.* Plasma beta-amyloid and white matter lesions in AD, MCI, and cerebral amyloid angiopathy. Neurology. 66, 23–29 (2006). 16401840 10.1212/01.wnl.0000191403.95453.6a

[b30] Verbeek M. M. *et al.* Cerebrospinal fluid amyloid beta(40) is decreased in cerebral amyloid angiopathy. Ann Neurol. 66, 245–249 (2009). 19743453 10.1002/ana.21694 PMC3697750

[b31] Grinberg L. T. & Thal D. R. Vascular pathology in the aged human brain. Acta Neuropathol 119, 277–290 (2010). 20155424 10.1007/s00401-010-0652-7 PMC2831184

[b32] Selnes P. *et al.* Effects of cerebrovascular disease on amyloid precursor protein metabolites in cerebrospinal fluid. Cerebrospinal Fluid Res. 7, 10 (2010). 20673341 10.1186/1743-8454-7-10 PMC2921347

[b33] Garcia-Alloza M. *et al.* Cerebrovascular lesions induce transient beta-amyloid deposition. Brain. 134, 3697–3707 (2011). 22120142 10.1093/brain/awr300 PMC3235567

[b34] Debette S. & Markus H. S. The clinical importance of white matter hyperintensities on brain magnetic resonance imaging: systematic review and meta-analysis. BMJ. 341, c3666 (2010). 20660506 10.1136/bmj.c3666 PMC2910261

[b35] Zhou Y., Yu F. & Duong T. Q. Alzheimer’s Disease Neuroimaging Initiative. White matter lesion load is associated with resting state functional MRI activity and amyloid PET but not FDG in mild cognitive impairment and early Alzheimer’s disease patients. J Magn Reson Imaging. 41, 102–109 (2015). 24382798 10.1002/jmri.24550 PMC4097981

[b36] Palop J. J. & Mucke L. Amyloid-beta-induced neuronal dysfunction in Alzheimer’s disease: from synapses toward neural networks. Nat Neurosci. 13, 812–818 (2010). 20581818 10.1038/nn.2583 PMC3072750

[b37] Hedden T. *et al.* Cognitive profile of amyloid burden and white matter hyperintensities in cognitively normal older adults. J Neurosci. 32, 16233–16242 (2012). 23152607 10.1523/JNEUROSCI.2462-12.2012 PMC3523110

[b38] Marchant N. L. *et al.* The aging brain and cognition: contribution of vascular injury and aβ to mild cognitive dysfunction. JAMA Neurol. 70, 488–495 (2013). 23400560 10.1001/2013.jamaneurol.405 PMC3771392

[b39] Marchant N. L. *et al.* Cerebrovascular disease, β-amyloid, and cognition in aging. Neurobiol Aging. 33, 1006.e25–36 (2012). 22048124 10.1016/j.neurobiolaging.2011.10.001 PMC3274647

[b40] Park J. H. *et al.* Effects of cerebrovascular disease and amyloid beta burden on cognition in subjects with subcortical vascular cognitive impairment. Neurobiol Aging. 35, 254–260 (2014). 23932881 10.1016/j.neurobiolaging.2013.06.026 PMC5849054

[b41] Lockhart A. *et al.* PIB is a non-specific imaging marker of amyloid-beta (Abeta) peptide-related cerebral amyloidosis. Brain 130, 2607–2615 (2007). 17698496 10.1093/brain/awm191

[b42] Ikonomovic M. D. *et al.* Post-mortem correlates of *in vivo* PiB-PET amyloid imaging in a typical case of Alzheimer’s disease. Brain 131, 1630–1645 (2008). 18339640 10.1093/brain/awn016 PMC2408940

[b43] Ly J. V. *et al.* 11C-PIB binding is increased in patients with cerebral amyloid angiopathy-related hemorrhage. Neurology 74, 487–493 (2010). 20142615 10.1212/WNL.0b013e3181cef7e3

[b44] Baron J. C. *et al.* Diagnostic utility of amyloid PET in cerebral amyloid angiopathy-related symptomatic intracerebral hemorrhage. J Cereb Blood Flow Metab. 34, 753–758 (2014). 24619277 10.1038/jcbfm.2014.43 PMC4013776

[b45] Brickman A. M. Contemplating Alzheimer’s disease and the contribution of white matter hyperintensities. Curr Neurol Neurosci Rep. 13, 415 (2013). 24190781 10.1007/s11910-013-0415-7 PMC3874404

[b46] Attems J. & Jellinger K. A. The overlap between vascular disease and Alzheimer’s disease—lessons from pathology. BMC Med. 12, 206 (2014). 25385447 10.1186/s12916-014-0206-2 PMC4226890

[b47] Kotagal V. *et al.* Modifiable cardiovascular risk factors and axial motor impairments in Parkinson disease. Neurology. 82, 1514–20 (2014). 24682965 10.1212/WNL.0000000000000356 PMC4011463

[b48] Toledo J. B. *et al.* Contribution of cerebrovascular disease in autopsy confirmed neurodegenerative disease cases in the National Alzheimer’s Coordinating Centre. Brain. 136, 2697–706 (2013). 23842566 10.1093/brain/awt188 PMC3858112

[b49] Vemuri P. *et al.* Vascular and amyloid pathologies are independent predictors of cognitive decline in normal elderly. Brain. 138, 761–71 (2015). 25595145 10.1093/brain/awu393 PMC4339775

[b50] Manjer J. *et al.* The Malmö diet and cancer study: Representativity, cancer incidence and mortality in participants and non-participants. Eur J Cancer Prev. 10, 489–499 (2001). 11916347 10.1097/00008469-200112000-00003

[b51] Folstein M. F., Folstein S. E. & McHugh P. R. “Mini-mental state”. A practical method for grading the cognitive state of patients for the clinician. J Psychiatr Res. 12, 189–198 (1975). 1202204 10.1016/0022-3956(75)90026-6

[b52] Petersen R. C. Mild cognitive impairment as a diagnostic entity. J Intern Med 256, 183–94 (2004). 15324362 10.1111/j.1365-2796.2004.01388.x

[b53] McKhann G. M. *et al.* The diagnosis of dementia due to Alzheimer’s disease: recommendations from the National Institute on Aging-Alzheimer’s Association workgroups on diagnostic guidelines for Alzheimer’s disease. Alzheimers Dement. 7, 263–269 (2011). 21514250 10.1016/j.jalz.2011.03.005 PMC3312024

[b54] Gelb D. J., Oliver E. & Gilman S. Diagnostic criteria for Parkinson disease. Arch Neurol. 56, 33–39 (1999). 9923759 10.1001/archneur.56.1.33

[b55] Schmidt P. *et al.* An automated tool for detection of FLAIR-hyperintense white-matter lesions in Multiple Sclerosis. NeuroImage. 59, 3774–3783 (2012). 22119648 10.1016/j.neuroimage.2011.11.032

[b56] Fazekas F., Chawluk J. B., Alavi A., Hurtig H. I. & Zimmerman R. A. MR signal abnormalities at 1.5 T in Alzheimer’s dementia and normal aging. AJR Am J Roentgenol. 149, 351–356 (1987). 3496763 10.2214/ajr.149.2.351

[b57] Wahlund L. O. *et al.* A new rating scale for age-related white matter changes applicable to MRI and CT. Stroke. 32, 1318–1322 (2001). 11387493 10.1161/01.str.32.6.1318

[b58] Wardlaw J. M. *et al.* STandards for ReportIng Vascular changes on nEuroimaging (STRIVE v1). Neuroimaging standards for research into small vessel disease and its contribution to ageing and neurodegeneration. Lancet Neurol. 12, 822–838 (2013). 23867200 10.1016/S1474-4422(13)70124-8 PMC3714437

[b59] Blennow K., Hampel H., Weiner M. & Zetterberg H. Cerebrospinal fluid and plasma biomarkers in Alzheimer disease. Nat Rev Neurol. 6, 131–144 (2010). 20157306 10.1038/nrneurol.2010.4

[b60] Koole M. *et al.* Whole-body biodistribution and radiation dosimetry of 18F-GE067: a radioligand for *in vivo* brain amyloid imaging. J Nucl Med. 50, 818–822 (2009). 19372469 10.2967/jnumed.108.060756

[b61] Lundqvist R. *et al.* Implementation and validation of an adaptive template registration method for 18F-flutemetamol imaging data. J Nucl Med. 54, 1472–1478 (2013). 23740104 10.2967/jnumed.112.115006

