# Supplementary material for: Cerebral white matter lesions – associations with Aβ isoforms and amyloid PET
Source: Sci Rep. 2016 Feb 9;6:20709. doi: 10.1038/srep20709 (PMC4746584; doi:10.1038/srep20709)
Supplement: Supplementary Information [file srep20709-s1.pdf]

Supplementary information

Cerebral white matter lesions – associations with Aβ isoforms and amyloid PET

Danielle van Westen, MD PhD[1, 2 *], Daniel Lindqvist, MD PhD[3, 4], Kaj Blennow, MD PhD[5, 6], Lennart Minthon, MD PhD[7, 8], Katarina Nägga, MD PhD[7, 8], Erik Stomrud, MD PhD[7, 8], Henrik Zetterberg, MD PhD[5, 9], Oskar Hansson, MD PhD[7, 8]

Supplementary Methods

Study population

The first sample was part of the prospective and longitudinal Swedish BioFINDER (Biomarkers For Identifying Neurodegenerative Disorders Early and Reliably) study (http://www.biofinder.se). Participants included 267 CHE and 360 patients with mild cognitive symptoms (MCS), i.e. patients with either subjective cognitive decline (SCD) or mild cognitive impairment (MCI). The CHE (103 males and 164 females; mean ± SD age: 72.9 ± 5.0) were recruited from the population-based Malmö Diet Cancer study.[1] Subjects were eligible for inclusion if they 1) were aged ≥ 60 years old, 2) scored 28-30 points on MMSE,[2] 3) did not suffer from any subjective cognitive impairment and 4) were fluent in Swedish. Exclusion criteria included presence of significant neurologic disease (e.g. stroke, PD, multiple sclerosis), severe psychiatric disease (e.g. severe depression or psychotic syndromes), dementia or MCI. Data were collected between 2009 and 2014 according to a standardized protocol.

The patients with MCS (180 males and 179 females; mean ± SD age: 70.7 ± 5.7) were enrolled consecutively at three memory outpatient clinics in Sweden and were thoroughly assessed by physicians with special interest in dementia disorders. The inclusion criteria were:

1) referred to the memory clinics due to cognitive impairment; 2) not fulfilling the criteria for dementia; 3) a Mini-Mental State Examination (MMSE) score of 24–30 points[2]; 4) age 60–80 years and; 5) fluent in Swedish. The exclusion criteria were: 1) cognitive impairment that without doubt could be explained by another condition (other than prodromal dementia); 2) severe somatic disease; 3) refusing lumbar puncture or neuropsychological investigation. MCS patients underwent thorough neuropsychological assessment with a large test battery under supervision of an experienced psychologist. Four broad cognitive domains were tested: Verbal ability (including A multiple-choice vocabulary test (SRB:1[3]) and Semantic Verbal Fluency (Condition 2, D-KEFS[4]), Episodic memory (including Rey Auditory Verbal Learning Test (RAVLT[5]), and Rey Complex Figure Test (RCFT[6])), Visuospatial construction ability (including Block design (WAIS[7]) and The copy trial of Rey Complex Figure Test), Attention and executive functions (including Trail Making Test (D-KEFS[4]) and Letter Verbal Fluency, Condition 1 (D-KEFS[4]). A senior neuropsychologist then stratified all patients into those with SCD (no measurable cognitive deficits) or MCI according to the consensus criteria for MCI suggested by Petersen. [8] In total 165 patients (46 %) were classified as SCD and 195 (54 %) patients as MCI.

A second sample comprised 110 subjects from a retrospective study, who all had been diagnosed with AD dementia the Memory Clinic at Skåne University Hospital, Malmö, between 2008 and 2014 (69 female, 41 male, mean ± SD age: 74.5 years ±7.3 years). At the initial visit, all patients were assessed by physicians experienced in dementia disorders, and underwent thorough physical, psychiatric and neurological examinations, as well as an interview that focused on their cognitive symptoms and ADL function. All patients met the dementia criteria and were diagnosed as probable AD according to NINCDS-ADRDA[9]; in addition, CSF Aβ42 was below 550 pg/L to confirm the presence of amyloid pathology.

The third sample, comprising 89 cases (34 female, 65 male; mean ± SD age: 65.4 years ± 11.2 years) with Parkinson´s disease (PD), henceforth referred to as the "PD cohort", is also part of the prospective and longitudinal Swedish BioFINDER (Biomarkers For Identifying Neurodegenerative Disorders Early and Reliably) study (www.biofinder.se). Participants were recruited at the Neurology and Memory Clinics. At the initial visit, all patients were assessed by physicians experienced in movement disorders and underwent thorough physical, psychiatric and neurological examination. PD diagnosis was set according to the NINDS Diagnostic Criteria.[10] Demographic characteristics of the cohorts are presented in Table 1.

Imaging

In the CHE, SCD and MCI subjects, MR imaging was performed at a 3 T Siemens Trio system and in the PD subjects at a 3 T Siemens Skyra system, equipped with standard head coils with 12 and 20 channels, respectively. Axial T2 FLAIR (27 and 23 slices, slice thickness 5.2 mm and 5 mm, respectively), coronal MPRAGE (180 slices, slice thickness 1.2 mm and 1 mm, image resolution 1 × 1 × 1.2 mm^3 and 1 × 1 × 1 mm^3, respectively) were acquired. In the AD subjects, imaging data included axial CT images (slice thickness 3-5 mm) in 96 cases and axial FLAIR images (slice thickness 5-6 mm at 1.5 T systems) in 14 cases.

White matter lesions (WML) and lacunes on MRI as well as WML and lacunes on CT were used as measures of SVD. For MR data in the CHE, SCD, MCI and PD cases, automated segmentation of WML was performed using the Lesion Segmentation Tool (LST) implemented in SPM8 (http://www.applied-statistics.de/lst.html). The LST segments WML in native space from a combination of high resolution T1-weighted and FLAIR images using a lesion growth algorithm.[11] The T1 image is segmented into three main tissue classes (CSF, GM and WM). This information is then combined with the FLAIR intensities in order to calculate lesion belief maps. By thresholding these maps by a user determined

threshold (kappa), an initial binary lesion map is obtained which is subsequently grown along voxels that appear hyperintense in the FLAIR image. The result is a lesion probability map. The disadvantage of this algorithm, the choice of the initial threshold kappa, is overcome by a routine using manual reference segmentations for a few images. Here, manual delineation of WML on FLAIR images, coregistered to the native high resolution T1 was performed in twelve individuals from this study, four controls, four MCI patients and four PD patients comprising. The manually segmented volume from these eight individuals ranged from 0.9 to 106.3 mL; the resulting optimal κ of 0.4 was used in the subsequent automated segmentation for all participants. The final result of the LST segmentation is a total lesion volume [mL], henceforth named 'WML volume', for each individual.

For MR data, visual rating of WML on FLAIR images was performed firstly according to the 3 point scale from Fazekas and colleagues.[12] Periventricular hyperintensity (PVH) was graded as 0=absence, 1=”caps” or pencil-thinning, 2=smooth "halo", 3=irregular PVH extending into the deep white matter. Separate deep white matter hyperintensities (DWMH) were rated as 0=absence, 1=punctate foci, 2=beginning confluence of foci, 3=large confluent areas. For statistical analysis, ratings of PVH and DWMH from the left and right hemispheres were summarized into total scores. Secondly, WML were assessed according to the ARWMC scale[13] that uses a similar 3-point scale. Each region, frontal, parieto-occipital and temporal, infratentorial and the basal ganglia, is rated separately and in addition a total score is calculated. WML are rated as absent=0, focal=1, beginning confluence=1, diffuse involvement =3. Lesions were included if the diameter was larger than 2 mm, except for the striatum, globus pallidus, and thalamus where the cut off diameter for lesion detection was 5 mm in order to separate WML from diffuse changes surrounding perivascular spaces that are frequently occurring in these areas. CT images in the AD subjects were assessed for WML

according to the Fazekas scale. The Fazekas and ARWMC scores based on MR images in the CHE, SCD and MCI subjects and on CT images in the AD subjects, correlated similarly (R^2=0.883 and R^2=0.871 respectively, Pearson correlation). For statistical analysis, scores from the left and right hemispheres were summarized.

The presence of lacunes was assessed according to Wardlaw et al.[14] using the FLAIR and MPRAGE sequences. Thus a lacune was defined as a round or ovoid, subcortical, fluid-filled cavity (signal similar to CSF) of 3 - 15 mm in diameter, on CT and often surrounded by a hyperintense rim on FLAIR, consistent with a previous acute small subcortical infarct or haemorrhage in the territory of one perforating arteriole. The total number of lacunes was recorded and this variable was dichotomized as lacunes present or absent.

Hippocampal volume and a scaling factor to correct for head size differences were determined in the CHE, SCD and MCI groups using Adaboost[15]; the mean of the left and right hippocampus multiplied by the scaling factor, was used in the statistical analysis. In the PD cohort, hippocampal and intracranial volume (ICV) were determined using VolBrain (http://volbrain.upv.es); here, the mean hippocampal volume was multiplied by the ICV to account for head size differences. In the AD group, the medial temporal lobe atrophy (MTA) score was determined and the mean score of the left and right MTA was used.[16]

^{18}F-Flutemetamol PET imaging and analysis

In 122 CHE, 101 SCD and 98 MCI subjects, the cerebral Aβ burden was measured using [^{18}F]-flutemetamol PET. Whole brain PET was scanning conducted at two sites using the same type of scanner, Philips® Gemini TF 16. Subjects received a single dose of [^{18}F]-flutemetamol according to a method described previously.[17, 18] The average uptake of [^{18}F]-flutemetamol was estimated by acquiring sum images 90-110 min post injection. Analysis was performed using the software NeuroMarQ, provided by GE Healthcare. Summed PET images were spatially normalized to Montreal Neurologic Institute (MNI) standard space

using a PET-only adaptive template registration method.[13] A volume of interest (VOI) template defined in MNI space was applied bilaterally in frontal, partietal, occipital and temporal VOIs, as well as in a composite VOI. The standardized uptake value ratio (SUVR) was defined as the tracer uptake in a VOI, normalized for the mean uptake in the cerebellar cortex, since this is free of fibrillar plaques.

Statistics

Logtransformation was performed of the WML volume, CSF levels of Aβ38, Aβ40 and Aβ42 as well as for the composite and regional SUVR for 18 F-flutemetamol. The distribution before and after logtransformation was checked by (i) visual inspection of the data using box-plots and histograms, (ii) comparing the mean and median for the individual variables, and (iii) comparing the distance between median and upper/lower quartile (Supplemental Table 1). After logtransformation, the composite and regional SUVR were less normally distributed.

Results:

Correlations between the independent and dependent variables, respectively

CSF levels of Aβ38 and Aβ40 did correlated slightly with CSF Aβ42 (r = 0.273 and 0.305, respectively, partial correlation, corrected for age, gender and hippocampal volume, data from the CHE, SCD and MCI groups). No significant correlation with the composite SUVR was found (r = 0.16 and 0.050, respectively). Only CSF Aβ42 showed a strong, inverse, correlation with the composite SUVR (r = -0.659). Thus, no variance attributable to AB42 of significant magnitude ($r > 0.7$) is shared with the other amyloid variables simultaneously included in the model.

The WML volume and total Fazekas score correlated strongly, as expected since these variables are different measurements of the abundance of white matter lesions (r = 0.752).

The correlation of these two with lacunes was only weak (r = 0.262 and 0.280, respectively) and thus no variance attributable to lacunes was shared by measures of white matter lesions.

Associations between the composite SUVR and SVD

Additional analysis was performed for the composite SUVR, now using binary regression with the 1.42 as cut-off for normalisation as previously described by Palmqvist et al [14]; results were similar for the logtransformed and dichotomized composite SUVR (Supplemental Table 1).

References:

1. Manjer J, et al. The Malmö diet and cancer study: Representativity, cancer incidence and mortality in participants and non-participants. *Eur J Cancer Prev.* **10,** 489-499 (2001).
2. Folstein MF, Folstein SE, McHugh PR. "Mini-mental state". A practical method for grading the cognitive state of patients for the clinician. *J Psychiatr Res.* **12**, 189-198 (1975).
3. Nilsson, LG et al. The betula prospective cohort study: Memory, health, and aging. *Aging Neuropsychol. Cogn.* **4,** 1–32 (1997).
4. Delis, DC, Kaplan E, Kramer, JH. *Delis-Kaplan executive function system TM: Examiner´s Manual.* (The Psychological Corporation A Harcourt Assessment Company, 2001).
5. Strauss E, Sherman EM, Spreen O. *A Compendium of Neuropsychological Tests. Administration, Norms, and Commentary – third edition*. (Oxford University Press, 2006).
6. Meyers JE, Meyers KR. *Rey Complex Figure Test and Recognition Trial.* (Psychological Resources, Inc., 1995).
7. Wechsler D. *Wechsler Adult Intelligence Scale - fourth edition. (Swedish version 2010. Stockholm, Psykologiförlaget AB. ed[SV1] .)*. (Harcourt Assessment, 2008).
8. Petersen RC. Mild cognitive impairment as a diagnostic entity. *J Intern Med* **256,** 183–94 (2004).
9. McKhann GM, et al. The diagnosis of dementia due to Alzheimer's disease: recommendations from the National Institute on Aging-Alzheimer's Association workgroups on diagnostic guidelines for Alzheimer's disease. *Alzheimers Dement.* **7**, 263-269 (2011).

10. Gelb DJ, Oliver E, Gilman S. Diagnostic criteria for Parkinson disease. *Arch Neurol.* **56**, 33-39 (1999).

11. Schmidt P, et al. An automated tool for detection of FLAIR-hyperintense white-matter lesions in Multiple Sclerosis. *NeuroImage.* **59**, 3774–3783 (2012).

12. Fazekas F, Chawluk JB, Alavi A, Hurtig HI, Zimmerman RA. MR signal abnormalities at 1.5 T in Alzheimer's dementia and normal aging. *AJR Am J Roentgenol.* **149**, 351-356 (1987).

13. Wahlund LO, et al. A new rating scale for age-related white matter changes applicable to MRI and CT. *Stroke.* **32**, 1318-1322 (2001).

14. Wardlaw JM, et al. STandards for ReportIng Vascular changes on nEuroimaging (STRIVE v1). Neuroimaging standards for research into small vessel disease and its contribution to ageing and neurodegeneration. *Lancet Neurol.* **12**, 822-838 (2013).

15. Morra JH, et al. Comparison of AdaBoost and support vector machines for detecting Alzheimer's disease through automated hippocampal segmentation. *IEEE Trans Med Imaging*. **29**, 30-43 (2010).

16. Scheltens P, et al. Atrophy of medial temporal lobes on MRI in "probable" Alzheimer's disease and normal ageing: diagnostic value and neuropsychological correlates. *J Neurol Neurosurg Psychiatry.* **55**, 967-972 (1992).

17. Blennow K, Hampel H, Weiner M Zetterberg H. Cerebrospinal fluid and plasma biomarkers in Alzheimer disease. *Nat Rev Neurol.* **6**, 131-144 (2010).

18. Koole M, et al. Whole-body biodistribution and radiation dosimetry of 18F-GE067: a radioligand for in vivo brain amyloid imaging. *J Nucl Med.* **50**, 818-822 (2009).

19. Lundqvist R, et al. Implementation and validation of an adaptive template registration method for 18F-flutemetamol imaging data. *J Nucl Med.* **54**, 1472-1478 (2013).

20. Palmqvist S, et al. Accuracy of brain amyloid detection in clinical practice using cerebrospinal fluid β-amyloid 42: a cross-validation study against amyloid positron emission tomography. JAMA Neurol. 2014;71:1282-9.

Supplemental Table 1. Associations between WML (volume and total Fazekas score) and SUVR from amyloid PET, as a continuous logtransformed variable as well as dichomotized as normal and abnormal.

		WML volume	Fazekas score
**Cognitively healthy elderly**	Composite SUVR	0.037	-0.137
	Composite SUVR (binary)	-0.010	-0.115
**Subjective cognitive decline**	Composite SUVR	0.099	-0.075
	Composite SUVR (binary)	0.080	0.053
**Mild cognitive impairment**	Composite SUVR	-0.028	-0.226
	Composite SUVR (binary)	-0.081	-0.20

Values represent standardized beta (linear regression). Values are corrected for age, gender and hippocampal volume; the latter was determined using Adaboost, the mean value of the left and right hippocampus was normalized using the scaling factor provided by this software to account for head size differences. Data on WML volume were not available for the AD group, where imaging mainly was performed using CT.
* = p<0.05, ** = p<0.01, *** = p>0.005
